# A genome-wide survey for prion-regulated miRNAs associated with cholesterol homeostasis

**DOI:** 10.1186/1471-2164-13-486

**Published:** 2012-09-17

**Authors:** Judith Montag, Markus Brameier, Ann-Christin Schmädicke, Sabine Gilch, Hermann M Schätzl, Dirk Motzkus

**Affiliations:** 1 German Primate Center, Unit of Infection Models, Kellnerweg, 4, 37077, Göttingen, Germany; 2 German Primate Center, Primate Genetics Laboratory, Kellnerweg 4, 37077, Göttingen, Germany; 3 Institute of Virology, Technical University of Munich (TUM), Trogerstrasse 30, D-81675, Munich, Germany; 4 Wyoming Excellence Chair in Prion Biology, Department of Veterinary Sciences and of Molecular Biology, University of Wyoming, 1000 E. University Ave, Laramie, USA

**Keywords:** Prion disease, Cholesterol, MicroRNA, Ultra-deep sequencing, Joint-profiling

## Abstract

**Background:**

Prion diseases are neurodegenerative diseases that are characterized by the conversion of the cellular prion protein (PrP^c^) into a pathogenic isoform (PrP^Sc^). It is known that neurodegeneration is often accompanied by the disturbance of cholesterol homeostasis. We have recently identified a set of genes that were upregulated after prion infection of N2a neuronal cells (Bach et al., 2009).

**Results:**

We have now used ultra-deep sequencing technology to profile all microRNAs (miRNA) that could be associated with this effect in these N2a cells. Using stringent filters and normalization strategies we identified a small set of miRNAs that were up- or downregulated upon prion infection. Using bioinformatic tools we predicted whether the downregulated miRNAs could target mRNAs that have been previously identified to enhance cholesterol synthesis in these cells. Application of this joint profiling approach revealed that nine miRNAs potentially target cholesterol-related genes. Four of those miRNAs are localized in a miRNA-dense cluster on the mouse X-chromosome. Among these, twofold downregulation of mmu-miR-351 and mmu-miR-542-5p was confirmed by qRT-PCR. The same miRNAs were predicted as putative regulators of the sterol regulatory element-binding factor 2 (Srebf2), the low-density lipoprotein receptor (Ldlr) or the IPP isomerase.

**Conclusions:**

The results demonstrate that joined profiling by ultra-deep sequencing is highly valuable to identify candidate miRNAs involved in prion-induced dysregulation of cholesterol homeostasis.

## Background

Transmissible spongiform encephalopathies (TSE) or prion diseases comprise a group of neurodegenerative diseases that include bovine spongiform encephalopathy (BSE) in cattle, scrapie in sheep, and Creutzfeldt-Jakob disease (CJD) in humans [1]. Prion diseases are associated with the conversion of the cellular prion protein (PrP^C^) into a pathogenic isoform (PrP^Sc^) [2,3]. Aggregation of PrP^Sc^ in neurons coincides with neuronal decay that finally leads to neurodegeneration. The molecular mechanism underlying prion-induced metabolic changes within infected cells are still poorly understood [4-6].

Several lines of evidence indicate that imbalances in the homeostasis of cholesterol are linked to the progression of neurodegenerative disorders including prion diseases [7-9]. Membrane-bound cholesterol is essential for the localization of PrP^C^ in lipid rafts and for the formation of PrP^Sc^[10]. Inhibition of cholesterol biosynthesis reduces the formation of PrP^Sc^ and prolongs the survival time of scrapie-infected mice [11-13]. Cell culture models have been widely used to identify molecules that link cholesterol synthesis to PrP^Sc^ formation [14]. Prion infected neuroblastoma cells upregulate a number of genes, among others HMG-CoA synthase (Hmgcs), HMG-CoA reductase (Hmgcr), IPP isomerase (Idi1), and the sterol regulatory element binding factor 2 (Srebf2), that are involved in the cholesterol biosynthesis pathway. In addition, expression of the low density lipoprotein (Ldl)-receptor is enhanced and leads to an increased uptake of cholesterol after prion infection [15].

Both, uptake and synthesis of cholesterol [16-20] as well as maintenance of hepatic function [21] are essentially influenced by the action of microRNAs (miRNAs). MiRNAs are potent regulators of gene expression that can act on the posttranscriptional level. These on average 22 nt long non-protein coding RNAs are derived from primary transcripts (pri-miRNA) that are sequentially processed into their mature form by the RNase III type nucleases DROSHA and DICER [22]. The mature miRNAs are sequestered into RNA-induced silencing complexes (RISCs) and recruit complementary mRNAs that, as a result, are degraded or translationally repressed [23]. Evidence accumulates that miRNAs are also involved in most neurodegenerative disorders that include Parkinsonism [24], Huntington’s Chorea [25], Alzheimer’s disease [26], and also prion disease [27,28].

Since miRNAs reduce the transcript levels of their target mRNAs on a genome-wide scale [29-33], the parallel examination of a miRNome and a transcriptome combined with a miRNA target analysis can be used to identify miRNA:mRNA interaction partners. This so-called “genome-wide joint profiling” has been successfully applied to identify pathologically relevant miRNA:mRNA interactions in Alzheimer’s disease [34] and neuronal HIV-infection [35].

In the present study we applied joint profiling to prion-infected neuronal cell cultures. Using ultra-deep sequencing we obtained several millions of small RNA sequence reads. Stringent filtering, co-expression and target analysis identified nine miRNAs that showed multiple targets in cholesterol-dependent mRNAs. Four of those miRNAs, namely mmu-miR-351, mmu-miR-503, mmu-miR-503*, and mmu-miR-542-5p, are clustered in a miRNA dense region on the mouse X-chromosome. Our results show that joint profiling of miRNA and mRNA is an effective approach to identify relevant miRNA:mRNA candidate pairs that could be used for functional studies in prion-induced dysregulation of cholesterol homeostasis.

## Methods

### RNA extraction

The mouse neuroblastoma cell line N2a (CCL-131, American type Culture Collection, Rockville, MD) was cultured and sibling clones were infected in parallel with brain homogenate from scrapie-infected mice (strain 22 L) or from healthy controls as previously described [15]. After the 18^th^ passage RNA was extracted with the RNeasy mini kit (Qiagen). The integrity of the RNA samples was determined by capillary electrophoresis on a Bioanalyzer (Agilent Technologies) according to the supplier’s instructions. The RNA concentration was determined photometrically at 260 nm on a NanoDrop (PeqLab) and 5 μg of each sample were applied to ultra-deep sequencing.

### Ultra-deep sequencing of small RNAs

Construction of cDNA libraries, cluster generation and subsequent ultra-deep sequencing on a Solexa/Illumina platform was performed at the Allan Wilson Centre Genome Service (AWCGS), New Zealand. In brief, 5 μg of total RNA from each sample was size-fractionated by 15% polyacrylamide electrophoresis (PAGE). The small RNA fraction (18–35 nucleotides (nt)) was extracted and ligated to 5’- and 3’-RNA adaptors using T4 RNA ligase. The RNA-adaptor constructs were purified and reverse transcribed. Clusters were generated by hybridization of the samples to the flow cell, chemical conversion to ssDNA, blocking of the 3’-OH and hybridization of sequencing primers. Ultra-deep sequencing was performed using one flow cell channel per sample.

### Bioinformatic analyses

Compression of library data was achieved by annotating the frequency of each unique sequence into its respective identifier, thereby removing multiple reads in FASTA format. Sequences with a frequency <10 were discarded. Both preprocessing steps were implemented as Perl scripts and led to a speed-up of all further computations by a factor of about 18. Processed libraries were aligned to miRBase release 14.0 with NCBI BLAST (blastall version 2.2.18) using standard settings without DUST filter (option -F F). Identification of miRNAs by BLAST required perfect alignment over the total miRNA length minus a tolerance offset of 3 nt. MiRNA target sites in the 3’-UTR of murine mRNAs Srebf2, Hgmcr, Hgmcs1, Mvk, Idi1, Fdft1, Cyp51, Sc4mol, and Ldlr were predicted using miRanda (version 3.0; [36]) with the following parameter settings: minimum score 130, minimum free energy −19 kcal/mol, conservation off, scaling parameter 4, gap open penalty −8.0, gap extend penalty −4.0. Ribosomal 28S, 18S, 5.8S and 5S rRNAs were BLASTed against miRBase release 14.0.

### miRNA profiling by quantitative real-time PCR

Ten ng of total RNA from scrapie- and mock-infected N2a cells were reverse transcribed (TaqMan MicroRNA Reverse Transcription Kit, Applied Biosystems). The relative expression levels of mmu-miR-503, mmu-miR-503*, mmu-miR-351 and mmu-miR-542-5p was determined in a stem-loop based quantitative real-time PCR (qRT-PCR) assay (TaqMan MicroRNA Assay, Applied Biosystems) according to the supplier's instructions. Cycling was performed after initial denaturation for 10 min at 95°C for 45 cycles with 95°C for 15 sec and 60°C for 45 sec in a 7500 Real Time PCR System (Applied Biosystems). Relative expression levels were calculated according to the ΔΔCT-method [37] using the non-regulated miRNA hsa-miR-106b* as a housekeeping RNA. Statistical significance was determined by two way ANOVA with Bonferroni post test (*** p < 0.001).

## Results

In the present study we applied joint profiling to prion-infected neuronal cell cultures. Therefore we performed co-expression analysis of miRNAs and mRNAs combined with a stringent miRNA target prediction to identify relevant miRNA:mRNA pairs. This combined approach will be referred to as joint profiling. Reliable joint profiling by a direct comparison of miRNA and mRNA expression data depends on the use of identical samples for both studies. Thus we have used the prion-infected neuroblastoma N2a cell model that had been generated for whole genome transcriptome profiling [15]. In brief, the N2a cells were infected in parallel with brain homogenate derived from terminally ill mice infected with the scrapie strain 22 L (ScN2a) or from mock-infected healthy mice (N2a-mock) as previously reported [15]. We then extracted total RNA from ScN2a and N2a-mock cells. To confirm high quality RNA we conducted nano-scale electrophoresis that showed a high RNA integrity number (RIN) of 9.4 and 9.6 in both samples, respectively.

The recent development of high-throughput sequencing technologies (also referred to as ultra-deep sequencing) allows for the comprehensive recording of all nucleic acid sequences in virtually any biological sample. We decided to use the Solexa platform that is especially suitable for miRNA analysis due to its immense capacity to generate high numbers of short sequence reads with high accuracy. To achieve the highest possible sensitivity both samples were enriched for small RNAs with a cut-off range from 18 to 35 nt. To further increase the sequencing depth (i.e. number of sequence reads per sample) we used one capillary per sample with a capacity of approximately 175 million base pairs. Our ultra-deep sequencing analysis resulted in the generation of 5.8 x 10^6^ and 4.2 x 10^6^ sequence reads from ScN2a and N2a-mock cells, respectively.

**Figure 1:**
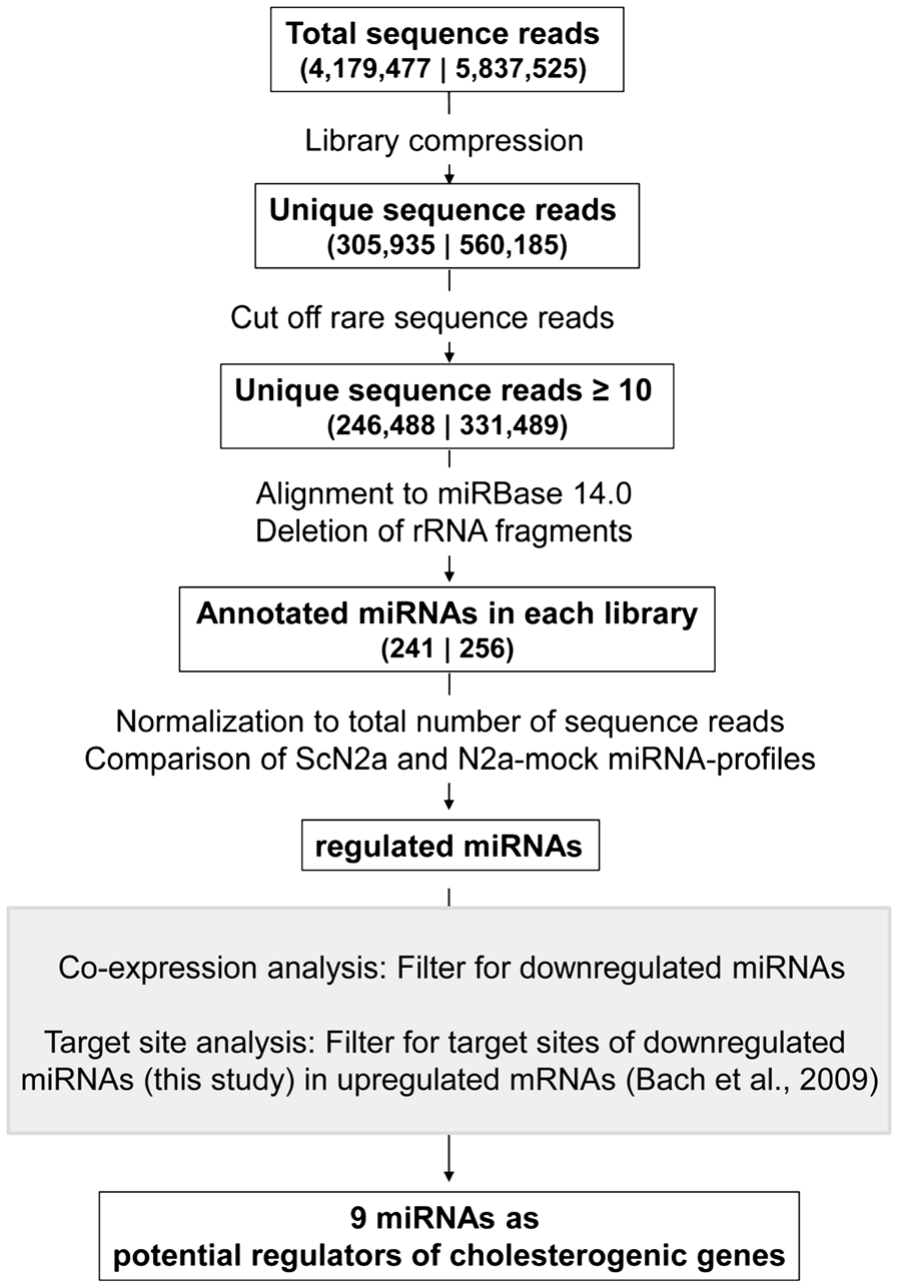
**Flow chart.** Successive filtering steps of ultra-deep sequencing reads from prion-infected ScN2a (left) and mock-infected N2a-mock (right) cells. Steps of the joint profiling approach are denoted in the shaded box.

For the generation of miRNA profiles from these libraries, successive filtering and normalization steps were implemented (Figure 1). As a first step, compression of data was achieved by merging of identical sequence reads and encoding their frequency into the respective sequence identifier. Since the population of small RNAs must be expected to include mRNA degradation products with limited significance [38] we increased the specificity of our dataset by exclusion of rare sequences. We arbitrarily applied a cut-off for reads with a frequency <10. It should be noted that this stringent cut-off results in loss of information, but is required to reduce the expected false positive rate. Identification of all expressed murine miRNAs in our samples was achieved by annotation of the filtered dataset to the miRBase 14.0. Our BLAST analysis allowed up to three mismatches at the 5’- or 3’-end of each miRNA, respectively. Although this leads to loss of specificity regarding closely related miRNAs, it enabled the identification of miRNA-processing variants that differ from the miRBase entries [39,40]. 

To date, several miRNAs have been removed from miRBase due to the fact that the sequences were identical to ribosomal RNAs (rRNAs), e.g. miR-560 and hsa-miR-923 from miRBase release 13.0 ( ftp://mirbase.org/pub/mirbase/13.0/miRNA.dead.gz). To ensure that our dataset would not include such rRNA-derived sequences, we did an additional BLAST analysis that compared the entire murine miRBase dataset with mouse 28S, 18S, 5S and 4.5S rRNA NCBI entries. We found that 16 miRNAs appeared to be rRNA fragments and thus were removed from our analysis. Most, but not all of these rRNA fragments were also described by others [41] and have meanwhile been deleted from mirBase. However, mmu-miR-709 and mmu-miR-1195 are identical to ribosomal sequences and still valid in the current mirBase release 19.

After this successive filtering we identified 256 miRNAs expressed in mock-infected and 241 expressed in scrapie-infected N2a cells, which collectively correspond to 261 individual miRNAs ( Additional file 1).

For the identification of regulated miRNAs, the libraries derived from scrapie-infected and mock-infected cells needed to be normalized. The normalization of ultra deep sequencing data has been reported to be a crucial step in interpreting regulation [38]. Parameswaran and colleagues have described two alternative methods, (1) the normalization to total small RNAs sequenced for each library or (2) the normalization against the total number of miRNAs in the respective samples. Applying both normalization methods to our filtered datasets we obtained similar results (data not shown). However, normalization against the total number of miRNAs bears the risk of creating a bias in case that a high proportion of miRNAs is regulated. Therefore we chose to normalize against the total number of sequence reads.

Next we aimed to identify miRNAs that were differentially expressed in scrapie-infected cells as compared to mock-infected controls. Regulation of miRNAs was considered to be robust if the relative abundance differed at least twofold between both libraries. Using this analysis strategy we found 14 upregulated and seven downregulated miRNAs in addition to a number of miRNAs that were solely expressed in ScN2a or N2a-mock cells. Since we had restricted our analysis to sequences that had been read more than ten times, we filtered the solely expressed miRNAs for those that were detected more than 20 times to reach the criterion of twofold regulation. Six miRNAs from N2a-mock cells matched this criterion and were termed “downregulated” without assigning a specific regulation factor. The resulting 14 upregulated and 13 downregulated miRNAs with their corresponding sequence reads are listed in Table 1.

**Table 1:** Regulated miRNAs in ScN2a cells as compared to mock infected controls

**miRNA**	**Number of reads**		**Regulation factor (normalized to total reads per library)**
	**N2a-mock**	**ScN2a**	
mmu-miR-351	88	0	**downregulated**
mmu-miR-139-5p	21	0	**downregulated**
mmu-miR-690	35	0	**downregulated**
mmu-miR-330*	36	0	**downregulated**
mmu-miR-378*	22	0	**downregulated**
mmu-miR-485*	27	0	**downregulated**
mmu-miR-375	65	11	**−4.23**
mmu-miR-503	64	12	**−3.82**
mmu-miR-203	47	10	**−3.37**
mmu-miR-542-5p	46	14	**−2.35**
mmu-miR-322*	33	11	**−2.15**
mmu-miR-503*	29	10	**−2.08**
mmu-miR-125b-3p	43	15	**−2.05**
mmu-miR-15b*	36	52	**2.02**
mmu-miR-598	83	120	**2.02**
mmu-miR-411	41	60	**2.04**
mmu-miR-743b-5p	47	69	**2.05**
mmu-miR-126-3p	70	106	**2.12**
mmu-miR-708	22	34	**2.16**
mmu-miR-883a-3p	10	16	**2.23**
mmu-miR-181d	184	295	**2.24**
mmu-miR-210	223	370	**2.32**
mmu-miR-497	13	22	**2.36**
mmu-miR-101a	12	21	**2.44**
mmu-miR-181c	33	61	**2.58**
mmu-miR-128	30	58	**2.70**
mmu-miR-338-5p	10	23	**3.21**

It is known that the upregulation of miRNAs results in the downregulation of their specific target genes. *Vice versa,* downregulation of miRNAs could lead to the upregulation mRNA levels, presumably by a de-repression mechanism [42]. Accordingly, some or all of the 13 downregulated miRNAs may be responsible for the upregulation of those mRNAs that induce prion-associated disturbance of cholesterol homeostasis.

In the light of these considerations we conducted a miRNA target search. We examined the 3’-UTRs of the cholesterogenic mouse genes (Srebf2, Hgmcs, Hgmcr, Mvk, Idi1, Fdft1, CYP51, Sc4mol, and Ldlr; [15]) for putative binding sites for the 13 downregulated miRNAs. Four out of 13 miRNAs were not predicted to bind any of the nine target genes. However, nine miRNAs showed multiple targets in at least one of these mRNAs (Table 2). Interestingly, four of those miRNAs, namely mmu-miR-351, mmu-miR-503, mmu-miR-503*, and mmu-miR-542-5p are located in a genomic cluster within 5 kb on the mouse X-chromosome. 

**Table 2:** Predicted target sites in the 3’-UTR of genes involved in the cholesterogenic pathway for downregulated miRNAs

**miRNA**	**Coordinates (NCBIM37)**	**Number of targets**							
		**Hgmcs1**	**Hgmcr**	**Idi1**	**Fdft1**	**CYP51**	**Sc4mol**	**Ldlr**	**Srebf2**
**mmu-miR-139-5p**	7: 108623890–108623957 [+]					1			
mmu-miR-330*	7: 19766814–19766911 [+]	1	2		1			2	
**mmu-miR-125b-3p**	9: 41390009–41390085 [+]			1	1	1		1	
**mmu-miR-690**	16: 28600021–28600129 [−]					1			
mmu-miR-378*	18: 61557489–61557554 [−]					2			
**mmu-miR-503**	X: 50407161–50407231 [−]			1	2			2	
mmu-miR-503*	X: 50407161–50407231 [−]		1						
**mmu-miR-351**	X: 50406432–50406530 [−]	1	1	1		1		5	3
**mmu-miR-542-5p**	X: 50402580–50402664 [−]			1					

**Figure 2:**
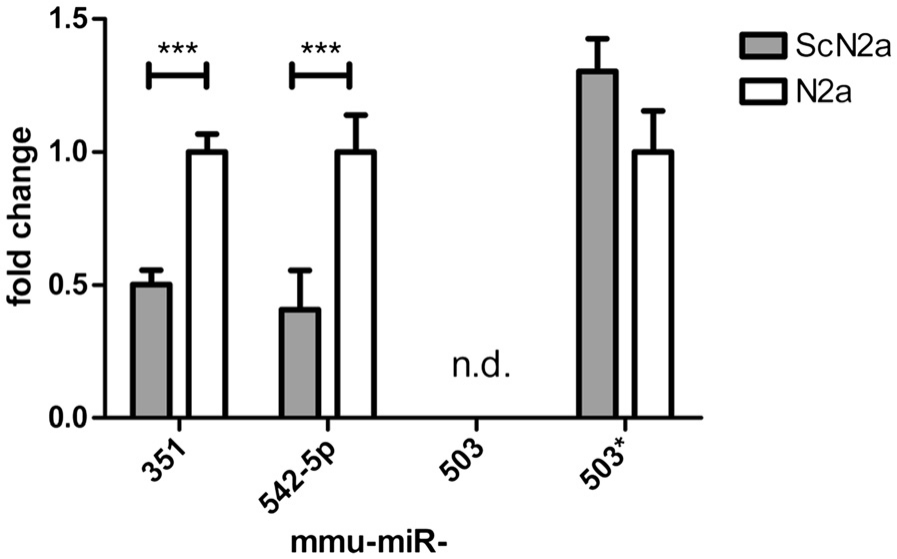
**qRT-PCR analysis of clustered miRNAs in scrapie-infected N2a cells.** Ten ng of total RNA from ScN2a and N2a-mock cells were applied to miRNA-specific cDNA synthesis. Subsequent qRT-PCR was performed using equivalent amounts of 1.3 ng RNA. Relative miRNA expression was analyzed by ΔΔC_T_ method [37] using the non-regulated miRNA mmu-miR-106b* as a housekeeping RNA and the non-infected cells as a calibrator. Statistical significance was determined by student-t-test (non-parametric with Welch’s correction, *** p < 0.001). The mean regulation factor ± SD of duplicates from three independent experiments are shown. Abbreviation n.d.; not detected.

We next validated the regulation of the miRNAs from this cluster (mmu-miR-351, mmu-miR-503, mmu-miR-503*, and mmu-miR-542-5p) by qRT-PCR. For normalization we used the mmu-miR-106b* as a housekeeping miRNA, presuming that it was expressed at constant and robustly high levels. Using a commercial kit, mmu-miR-503 was not detected by qRT-PCR analysis. To provide comparability among the quantification of the different miRNAs we did not modify the recommended protocol of the commercial detection kit in order to achieve detection of mmu-miR-503. In comparison, mmu-miR-503* could be detected, however, we could not confirm regulation. The fact that in ultra-deep sequencing the number of sequence reads and the upregulation factors were both at the borderline may have prevented the robust detection of miR-503*-regulation by qRT-PCR. However, the regulation of the more abundantly expressed miRNAs mmu-miR-351 and −542-5p was confirmed by qRT-PCR, indicating to a high validity of the ultra-deep sequencing (Figure 2). Furthermore the magnitude of downregulation in scrapie-infected versus mock-infected cells of mmu-miR-351 and mmu-miR-542-5p was comparable to that estimated by ultra-deep sequencing. In summary, our joint profiling approach lead to the identification of two miRNA candidates that correlate with disturbance in cholesterol homeostasis in prion-infected cells.

## Discussion

Ultra-deep sequencing represents a powerful technology for the comprehensive assessment of the whole miRNome in a given sample in a presumably unbiased manner. Using Solexa technology we have generated five million independent short sequence reads per sample. Highly abundant miRNAs, such as mmu-let-7c, mmu-miR-24, and mmu-miR-99b were sequenced on average 20,000 times. This corresponds to an enhanced sensitivity of three orders of magnitude in comparison to traditional cloning and subsequent Sanger sequencing strategies in early pioneer studies [43]. Since high sensitivity mostly results in reduced specificity we used a panel of stringent filters to identify miRNAs that were robustly expressed. Contemporaneously we could exclude fragments derived from ribosomal RNA that were misleadingly annotated as miRNAs in miRBase 14.0. The high specificity of our approach is reflected by the detection of more than 1,000 sequence reads of the neuronally expressed paralogues miR-103/107 [44] and miR30a/d [45], whereas liver-enriched miR-122 was not expressed in N2a cells. This is in accordance with previous studies that did not detect significant amounts of these miRNAs in the brain [28,43,46].

Ultra-deep sequencing data can be used for relative quantification, but the resulting accuracy is strictly dependent on the used scaling method [47]. We have normalized our miRNAs expression profiles to the respective library size as suggested [48]. To ensure the applicability of this normalization method to identify regulated miRNAs we performed qRT-PCR. By comparison of these two independent methods we could confirm downregulation of mmu-miRs −351 and −542-5p. Furthermore, the observed differences in miRNA expression were consistent, regardless whether they were determined by UDS or qRT-PCR, although significant regulation of one miRNA could not be confirmed.

It was recently reported that murine mmu-miRNA-146a is upregulated in scrapie [27], and BSE-infected [49] mice. miR-146a can be activated upon LPS treatment via Toll-like receptors TLR2 and TLR4. Upon overexpression, mmu-miR-146a modulates transcription of genes involved in cell movement and adhesion, mediators of inflammation and a set of genes involved in RNA post-transcriptional regulation [49]. Comparison of N2a vs ScN2a cells revealed a 1.6 fold upregulation of mmu-miR-146a that failed to match our criteria for further analysis. This is similar to the regulation upon BSE-infection in macaques [28]. It may also reflect the specificity of certain microRNAs in rare human prion diseases that was recently reported [50].

Our approach revealed 14 upregulated and 13 downregulated miRNAs in ScN2a cells as compared to mock-infected controls. The downregulated miRNAs were used as queries for target analysis to identify mutual miRNA:mRNA interaction partners that might be responsible for prion-associated induction of cholesterol synthesis. Linking a miRNA to its downstream targets is a major challenge in miRNA biology and only few miRNA:mRNA pairs have been experimentally confirmed yet. A variety of bioinformatic algorithms have been developed which predict potential binding sites within the 3’-untranslated regions (UTRs) of mRNAs [51]. Some, but not all limitations of miRNA target predictions are caused by the inaccuracy of the underlying algorithm. Identification of relevant miRNA:mRNA pairs can also fail if one, the miRNA or the mRNA, is not present in the relevant cell or tissue. Using our miRNA expression profile and the known mRNA expression profile of the N2a cells we could strongly limit the assumed production of false positive or false negative results. We used the miRanda algorithm [36] to predict putative binding sites of the downregulated miRNAs in the 3’-UTRs of prion-induced mRNAs related to cholesterol homeostasis [15].

Four downregulated miRNAs could not be linked to the upregulated cholesterogenic genes. These miRNAs have neither been described to be involved in cholesterol homeostasis nor in prion disease. However, nine out of the 13 downregulated miRNAs were predicted to have at least one target site in the 3’-UTR of the examined mRNAs. Notably four of these miRNAs reside within a 6 kb cluster on the mouse X-chromosome. To our knowledge, this is the first time that this cluster is correlated to neurogenic cholesterol metabolism. A putative function of the homologous microRNA cluster in humans has been recently described. The concerted regulation of cluster-derived miRNAs promoted cell cycle arrest and differentiation in myocytes [52] and in monocytes [53]. Co-expression of clustered miRNAs that results in cooperative function has been discovered in several biological systems such as development [54], stem cell differentiation [55], or viral infection [56]. Interestingly we also identified two upregulated miRNAs, mmu-miR-883a-3p and mmu-miR-743b-5p, that were localized on a second X-chromosomal cluster. Additional five miRNAs that reside in this cluster did not match our stringent criteria, but were upregulated more than 1.5 fold. Whether regulation of these clusters is a cause or a consequence of prion infection remains unknown. N2a is a polyploid cell line with a mean number of chromosomes around 100 and an unstable chromosomal content upon passaging and even within the same culture [57]. Whether or not the expression of the X-chromosomal miRNA cluster is also influenced by the used cell culture conditions cannot be estimated.

It should be noted that the expression of miRNAs are highly cell- and tissue-specific. Thus differences in miRNA expression between prion-infected and non-infected N2a cells may not reflect miRNA dysregulation in brains of prion-diseased mice or humans. Consequently, the question whether our findings may also have an indication for human prion diseases cannot be answered by our study. However, since imbalances in neuronal cholesterol homeostasis plays a pivotal role in several human neurodegenerative diseases, including Alzheimer's disease [58], Huntington’s chorea [59,60], and Niemann Pick disease type C [61] our results may be useful to elucidate the mechanism that underlies cholesterol dysregulation. Future studies that will focus on the identification of putative targets of miR-351 and miR-542-5p need to be carried out to reinforce the significance of our results.

## Conclusion

We have identified a number of regulated miRNAs in a cell culture model for prion diseases by the use of ultra-deep sequencing. Using a joint profiling approach we found that the X-chromosomal clustered mmu-miRs-351 and 542-5p could target genes that are involved in prion-induced dysregulation of cholesterol homeostasis. Our results show that semi-quantitative analysis of ultra-deep sequencing data is capable to identify differentially regulated miRNAs from biological samples.

## Competing interests

The authors declare that they have no competing interests.

## Authors’ contributions

DM designed research, MB performed bioinformatics, HSM and SG produced and provided samples, ACS performed qRT-PCR, DM and JM wrote the manuscript with contributions from all authors. All authors read and approved the final manuscript.

## Supplementary Material

Additional file 1 miRNA expression profiles of ScN2a and N2a-mock cells. Click here for file
